# Expression and Tumor-Promoting Effect of Tyrosine Phosphatase Receptor Type N (PTPRN) in Human Glioma

**DOI:** 10.3389/fonc.2021.676287

**Published:** 2021-09-07

**Authors:** Dong Wang, Fan Tang, Xi Liu, Yueshan Fan, Yu Zheng, Hao Zhuang, Budong Chen, Jie Zhuo, Bo Wang

**Affiliations:** ^1^Department of Neurosurgery, Tianjin Medical University, General Hospital, Tianjin Key Laboratory of Injuries, Variations, and Regeneration of Nervous System, Tianjin Neurological Institute, Tianjin, China; ^2^Department of Pathology, Tianjin Huanhu Hospital, Tianjin Key Laboratory of Cerebral Vascular and Neurodegenerative Diseases, Tianjin Neurosurgical Institute, Tianjin, China; ^3^Department of Gastroenterology, Tianjin Nankai Hospital, Tianjin, China; ^4^GCP Center, Tianjin Medical University Cancer Institute & Hospital, Tianjin, China; ^5^Department of Hepatic Biliary Pancreatic Surgery, Cancer Hospital Affiliated to Zhengzhou University, Zhengzhou, China; ^6^Department of Neurosurgery, Tianjin Huanhu Hospital, Tianjin Key Laboratory of Cerebral Vascular and Neurodegenerative Diseases, Tianjin Neurosurgical Institute, Tianjin, China; ^7^Department of Neurosurgery, Tianjin University Huanhu Hospital, Tianjin, China; ^8^Department of Neurosurgery, Huanhu Hospital Affiliated to Nankai University, Tianjin, China

**Keywords:** PTPRN, glioma, RNA-seq, GSEA, Akt

## Abstract

Tyrosine phosphatase receptor type N (PTPRN) plays an important role in the regulation of the secretion pathways of various neuroendocrine cells. Moreover, PTPRN was demonstrated to play a crucial role in the initiation and progression of the signalling cascade regulating cell function. In this study, fifty-seven glioma patients were enrolled for clinical and prognostic analyses. The cell phenotype was determined by cell proliferation and migration assays. RNA-seq, co-IP and mass spectrometry were used to study the molecular mechanism of the effects of PTPRN on cell proliferation and metastasis. The result showed that High expression of PTPRN indicated a poor prognosis of high-grade glioma. PTPRN downregulation reduced the proliferation and migration of glioma cells, and PTPRN overexpression induced the proliferation and migration of glioma cells. PTPRN knockdown decreased tumor growth in a mouse xenograft model. Effect of PTPRN knockdown on the transcriptome was studied in U87 glioma cells. PTPRN activated the PI3K/AKT pathway by interacting with HSP90AA1. In conclusion, PTPRN is an important proliferation- and metastasis-promoting factor. Reducing the expression of PTPRN in glioma cells can be used as a potential therapeutic strategy.

## Introduction

Glioma accounts for approximately 50% of all malignant brain tumors (1). Prognosis of glioma patients is related to the volume, WHO grade and location of the tumor. Currently, radiotherapy, chemotherapy, gene therapy and other treatment methods are mainly used in the clinic. However, due to chemoradiotherapy resistance, the median survival time of high-grade glioma patients is less than 12 months (2). Therefore, urgent investigations of the molecular mechanism of occurrence and development of gliomas and exploration of effective methods for the diagnosis and treatment of glioma are needed.

Protein tyrosine phosphatase receptor type N (PTPRN) is located on human chromosome band 2q35. PTPRN is mainly expressed in endocrine cells, neurons of autonomic nervous system and neuroendocrine neurons of brain, including pancreas, pituitary, adrenal medulla, amygdala and hypothalamus, because they all contain neurosecretory granules (3, 4). PTPRN is a type I transmembrane protein that has an inactive protein tyrosine phosphatase (PTP) domain (5). PTPRN participates in neuroendocrine processes, such as biogenesis, transport and/or regulation of exocytosis (6). Torii S et al. demonstrated that PTPRN has an important effect on the regulation of the secretion pathways in various neuroendocrine cells, possibly by regulating hormone content (7). Diabetes-related gene screening experiments indicated that PTPRN plays an important role in occurrence and development of diabetes mellitus (8–10). Mziaut H demonstrated that PTPRN increases secretory granule gene transcription by binding to STAT5 (11).

We analysed the prognosis of glioma patients and observed the effect of PTPRN on the phenotype of glioma cells. To clarify the mechanism of action of PTPRN in glioma cells, we downregulated PTPRN, detected the changes in the pathways in glioma cells, and determined possible factors related to PTPRN that influenced the changes in the pathways.

## Materials And Methods

### Clinical Specimens

Retrospective analysis of 57 patients with glioma. The patients did not undergo any treatment before surgery. The control brain tissue of 5 cases (cerebral cortex) were from the decompressor brain tissue which were removed by trauma patients. All clinical tissue samples were taken from Tianjin Huanhu hospital. All the specimens were obtained with the consent of the ethics committee of Tianjin Huanhu hospital and the consent of the patients’ family members. The WHO grades of glioma patients were assigned by two pathologists, and they were followed up for 3 years.

### The Cancer Genome Atlas (TCGA) Dataset

TCGA database was analyzed by “Gene Expression Profiling Interactive Analysis (GEPIA)” and “The Human Protein Atlas”. GEPIA provides key interactive analysis and customization functions, including tumor/normal differential expression profile analysis, profiling, pathological staging, patient survival analysis, similar gene detection analysis, and dimensionality reduction analysis. GEPIA was used to analyze the PTPRN expression in GBM of TCGA, and the survival analysis module was used. The screening conditions were as follows, method: OS, group cut off: median, hazards ratio: yes, 95% confidence interval: yes, axis units: months; data sets selection: GBM. The Human Protein Atlas is to study the immunohistochemical staining status of proteins in human normal tissues, cancer tissues, and cancer cell lines. It can be used to analyze the differential expression of proteins in tumor tissues and to analyze the survival of genes and tumors. The screening conditions were as follows: pathology, prognostic summary, cut off: median.

### Immunohistochemical Analysis

Immunohistochemistry was used to detect the expression of PTPRN, vascular endothelial growth factor (VEGF), and O6-methylguanine-DNA methyltransferase (MGMT). The operation steps were strictly in accordance with the instructions of the kit. 4μM thick sections were dewaxed with xylene. Gradient ethanol dehydration. Citrate buffer (pH=6.0) was used for antigen repair in a pressure cooker. After cooling, PBS was washed 3 times. First antibody: anti-PTPRN antibody (ab207750, 1:200, Abcam, UK), anti-VEGF antibody (EP1176Y, 1:200, Zhongshan, China), anti-MGMT antibody (UMAB56, 1:200, Zhongshan, China), were added and incubated at 4°C overnight. After the first antibody was rewarming and washed with PBS, the second antibody (DS9800, Leica, German) was added and incubated at room temperature for 20 min. DAB stained, hematoxylin stained, and neutral resin was used to seal the sections.

Each section was determined by two senior pathologists according to the staining proportion and intensity of positive cells. According to the staining intensity, no staining score was 0, the light yellow (weak positive) score was 1, the brown-yellow (medium positive) score was 2, and the brown (strongly positive) score was 3. According to the staining proportion of positive cells, the score of no positive tumor cells was 0, the score of positive cells less than 20% was 1, the score of 20% to 50% was 2, the score of 51% to 70% was 3, and the score of more than 70% was 4. Multiply the above two scores as the final result.

### Cell Culture and Transfection

We used the U87 and U251 glioma cell lines (ATCC) in the present study. The cells were cultured in a DMEM medium containing 10% FBS at 37°C and 5% CO_2_. The cells were transfected when they reached the logarithmic growth phase. For cell transfection to downregulate and upregulate PTPRN, short-hairpin RNA and overexpression plasmids were used, respectively. The oligonucleotides targeting PTPRN were ligated to pLKO.1 vector, following the procedure of Addgene, and the vector with a similar GC ratio and no random sequence matched with any known human coding gene was ligated as the control group. The overexpression plasmid was constructed using cDNA of the human U87 glioma cell line as a template. PCR products were ligated to the TA clone vector PCR8/GW/TOPO (Invitrogen, USA). The recombinant plasmid containing the correct fragment was recombined with lentivirus target plasmid by LR clonase using gateway method. (**Supplementary Table 1**).

### Reverse Transcription-Quantitative Polymerase Chain Reaction (RT-qPCR)

Total RNA was extracted with RNase-free reagent. TRIzol (Invitrogen, USA) was used for RNA isolation, which was followed by RNA reverse transcription. RNA precipitation was dissolved in pure water without RNase, and the concentration of RNA was determined by spectrophotometer (A260/280). RT-qPCR was performed using an SYBR kit (Promega, USA), and the results were calculated by the 2^-△△Ct^ method. The primers for PTPRN and GAPDH used in this study are shown in the **Supplementary File**.

### Western Blot

The protein was extracted with RIPA lysis buffer, and the concentration of the protein was assayed by the BCA method. Proteins were denatured at 100°C for 15 min and separated by 10% SDS-PAGE. PVDF membranes were blocked with 5% BSA (Solarbio, China) and incubated with primary antibodies against PTPRN (ab207750, 1:1000, Abcam, UK), E-cadherin (3195S, 1:1000, CST, USA), N-cadherin (13116S, 1:1000, CST, USA), Snail (3879S, 1:1000, CST, USA), HSP90AA1(8165S, 1:1000, Abcam, UK), AKT (3195S, 1:1000, CST, USA), p-AKT (4060S, 1:1000, CST, USA), mTOR (2983S, 1:1000, CST, USA), p-mTOR (2974S, 1:1000, CST, USA), GAPDH (5174S, 1:1000, CST, USA) and secondary antibodies (7074S, 1:2000, CST, USA). The expression of these proteins was visualized by using an enhanced chemiluminescence kit (Merck, USA).

### Cell Counting Kit-8 Assay

The cell counting kit-8 (CCK-8, Beyotime, China) assay was used to measure the proliferation of the cells. In 96 wells plate, 1×10^3^ cells were added into each well, and each group of cells was set with 3 multiple wells. When testing was needed, a CCK-8 solution was added. After incubation in an incubator for 2 h, the absorbance value at 450 nm was detected by spectrophotometer. The corresponding OD values were detected at 24 h, 48 h, 72 h, and 96 h. The experiment was repeated three times.

### Colony Formation Assay

The colony formation assay was used to measure the proliferation of the cells. In 96 wells plate, cells transfected with PRPTN-shRNA and PTPRN plasmids were cultured for 14 days under standard conditions at 1×10^3^ cells/well. Then, the colonies were fixed with 4% paraformaldehyde and stained with 0.5% crystal violet. The results were recorded using a camera. The experiment was repeated three times.

### Wounding Healing Assay

The wounding healing assay was used to measure the migration of the cells. Cells transfected with PRPTN-shRNA and PTPRN plasmids were cultured in 6-well plates. The cells at a density of 5×10^4^/well were scratched with a pipette tip. Then, the cells were cultured in a serum-free medium for 24 h. The scratch was imaged at 0 and 24 h. The area of scratch reduction was calculated. The experiment was repeated three times.

### Extreme Limiting Dilution Analysis

The extreme limiting dilution analysis was used to measure the self-renewal ability of glioma stem cells. Cells transfected with PRPTN-shRNA and PTPRN plasmids were cultured in DMEM/F12 with B27 (Gibco, USA), insulin, EGF, and bFGF (Sino, China) for 14 days. The glioma stem cell (GSC) frequency was calculated by using extreme limiting dilution analysis (ELDA, bioinf.wehi.edu.au/software/elda/).

### RNA-Sequencing

The efficiency of transfection was verified by reverse transcription-quantitative polymerase chain reaction. The U87 cells and PTPRN knockdown U87 cells were lysed with TRIzol. Total RNA was extracted with RNase-free reagent. RNA-seq was performed by BGI Company (Shenzhen, China) using BGISEQ-500. GEO Series record number was GSE175831. We also carried out gene set enrichment analysis (GSEA, USA) and pathway enrichment analysis.

### Co-Immunoprecipitation (Co-IP) and Liquid Chromatography-Tandem Mass Spectrometry (LC-MS/MS) Analysis

Transfected 293T or U87 cells were collected, and lysis buffer (20 mM Tris-HCl (pH 8), 137 mM NaCl, 0.5% Triton X-100, and 2 mM EDTA) and protease inhibitor cocktail were added at 4°C for 30 min. The mixture was centrifuged at 15,000rpm for 30 min. Part of the supernatant for Co-IP was incubated with a primary anti-FLAG antibody (Sigma-Aldrich, USA) overnight. The agarose slurry was added and slowly shaken for 4 h at 4°C. Then, the agarose slurry was collected, and the adsorbed protein was washed and collected for LC-MS/MS analysis (Shanghai Applied Protein Technology) and western blot analysis.

### Animal Experiments

The animal experiment design was approved by the animal experiment ethics review committee of Tianjin Huanhu hospital. All operations and experimental procedures are in accordance with the regulations on the management of laboratory animals. The experimental animals were reared in the SPF animal laboratory of the experimental animal center of Tianjin Medical University. Twelve 5-7 weeks old athymic nude mice weighing 17-20 g (Huafukang Biotechnology, China) were randomly divided into the control group and PTPRN knockdown group. The nude mice were anesthetized and placed in a single-arm digital display stereotactic device. A skull drill was drilled 1 mm in front of the anterior fontanel and 2 mm on the right side of the midline. Then, 1× 10^5^ cells were injected into the brain. After that, the motor ability and neurological signs of the mice were observed every 3 days and weighed once a week. The nude mice were euthanized when they showed signs of cachexia or neurological dysfunction. The mice were imaged for fluc activity by a bioluminescence imaging system (IVIS^®^ Lumina III, USA) and examined by magnetic resonance imaging (MRI, Siemens, German).

### Statistical Analysis

The data of the study were analysed by SPSS 16.0 (Chicago, IL, USA). The data are presented as the mean ± SD. The clinical data were analysed by ANOVA. The survival of patients and nude mice was analysed by the Kaplan-Meier method. The T test was used for comparison of two groups. p<0.05 indicated that the results are statistically significant.

## Results

### High Expression of PTPRN Indicated a Poor Prognosis of High-Grade Glioma

**Figure 1:**
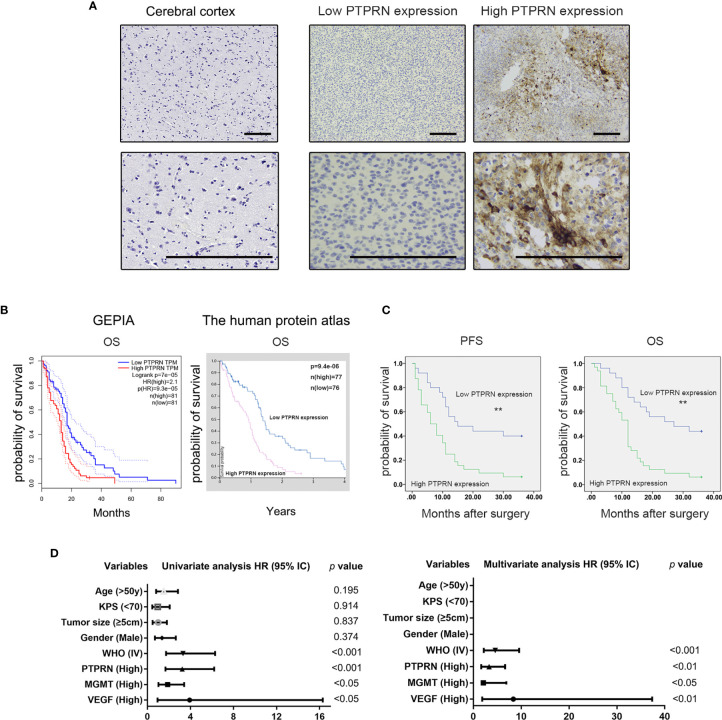
High expression of PTPRN indicated a poor prognosis of high-grade glioma. **(A)** PTPRN expression detected by immunohistochemistry staining in cerebral cortex tissue (negative) and glioblastoma (low and high PTPRN expression); **(B)** Survival analysis of PTPRN expression based on the GEPIA and Human Protein Atlas database (log-rank test); **(C)** Survival analysis of PTPRN expression in fifty-seven high-grade glioma patients (log-rank test); **(D)** Univariate and multivariate survival analysis between PTPRN expression and clinicopathological parameters; scale bars, 100 μm; **p < 0.01.

Survival analysis of PTPRN expression based on the TCGA database showed that high expression of PTPRN indicated a poor prognosis of high-grade glioma (**Figure 1B**). Our clinical and experimental results were consistent with the database analysis results (**Figure 1C**). The expression level of PTPRN was divided into a low (score 0-4) and high (score 6-12) expression group using the mean expression score 5.28 as a cutoff point (**Figure 1A**). The overall survival (OS) of patients with high PTPRN expression was shorter than that of patients with low PTPRN expression. Univariate analysis showed that the level of PTPRN, MGMT, VEGF and WHO grade were associated with the prognosis of glioma patients. Moreover, the expression of PTPRN was an independent factor for the prognosis of glioma patients (**Figure 1D**). In addition, we found that there was a positive correlation between PTPRN and MGMT; however, there was no association between the level of PTPRN and the other clinicopathological indexes, which include sex, age, Karnofsky Performance Status (KPS), tumor diameter, VEGF (**Table 1**).

**Table 1:** The relationship between PTPRN expression with clinicopathological features.

Clinicopathological	Patients	PTPRN (Mean value )		P value
features	(n)	Low (n)	High (n)	
**Sex**				0.775
Male	18	7	11	
Female	39	18	21	
**Age**				0.585
<50y	22	11	11	
≥ 50y	35	14	21	
**WHO grade**				0.792
III	28	13	15	
IV	29	12	17	
**Tumor size**				1.000
<5 cm	41	18	23	
≥ 5 cm	16	7	9	
**Karnofsky**				0.308
<70	47	19	28	
≥ 70	10	6	4	
**MGMT**				0.033
Low	29	17	12	
High	28	8	20	
**VEGF**				1.000
Low	5	2	3	
High	52	23	29	

### PTPRN Downregulation Reduced the Proliferation and Migration of Glioma Cells

**Figure 2:**
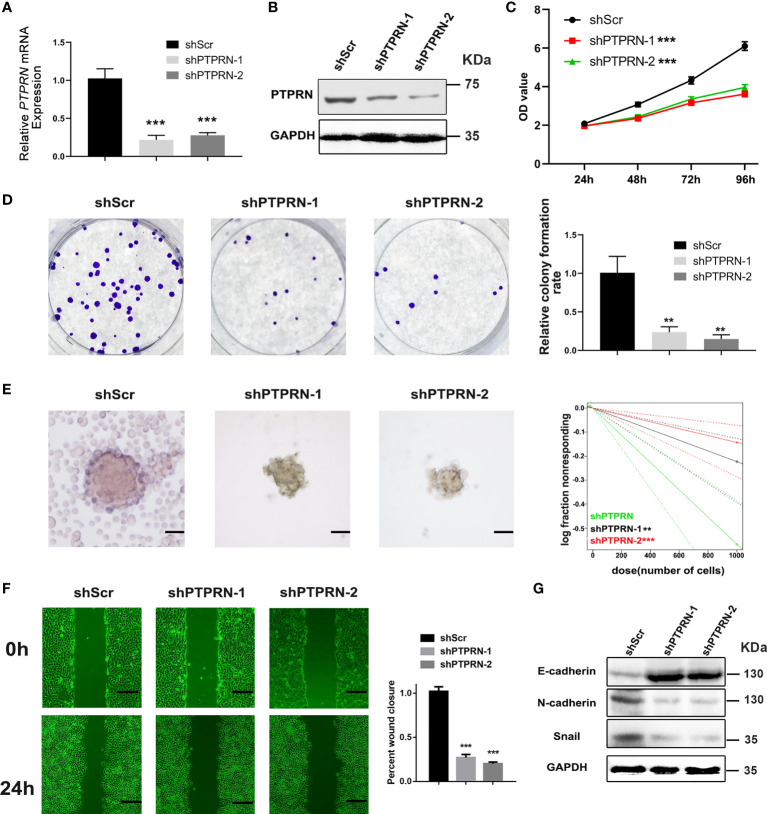
PTPRN downregulation reduced the proliferation and migration of U87 cells. **(A, B)** The level of PTPRN was assayed by RT-qPCR and western blot analysis after transfection with PTPRN-shRNA; **(C)** The proliferation of the cells was detected by CCK-8; the corresponding OD values were detected at 24 h, 48 h, 72 h and 96 h; **(D)** The proliferation of the cells was detected by colony formation assays; the results were recorded using a camera at 14 days; **(E)** Sphere formation detected by microscope and calculated by ELDA after 14 days; **(F)** The migration of the cells detected by wound healing assay; the scratch was imaged at 0 and 24 h; The area of scratch reduction was calculated; **(G)** EMT-related protein (E-cadherin, N-cadherin, and Snail) expression in shPTPRN U87 cells; scale bars, 100 μm; **p < 0.01, ***p < 0.001.

The level of PTPRN was measured by RT-qPCR and western blot analysis after the transfection with PTPRN-shRNA (**Figures 2A, B**). The results of CCK8 and colony formation assays showed that knockdown of PTPRN reduced the proliferation of U87 cells (**Figures 2C, D**). The migration was inhibited in U87 cells with low expression of PTPRN (**Figure 2E**). The glioma stem cell frequency was decreased in the PTPRN knockdown group (**Figure 2F**). The detection of epithelial mesenchymal transition (EMT)-related markers by western blot analysis showed that N-cadherin and Snail expression followed the trend of PTPRN. However, the level of E-cadherin was increased by knockdown of PTPRN (**Figure 2G**).

### PTPRN Overexpression Induced the Proliferation and Migration of Glioma Cells

**Figure 3:**
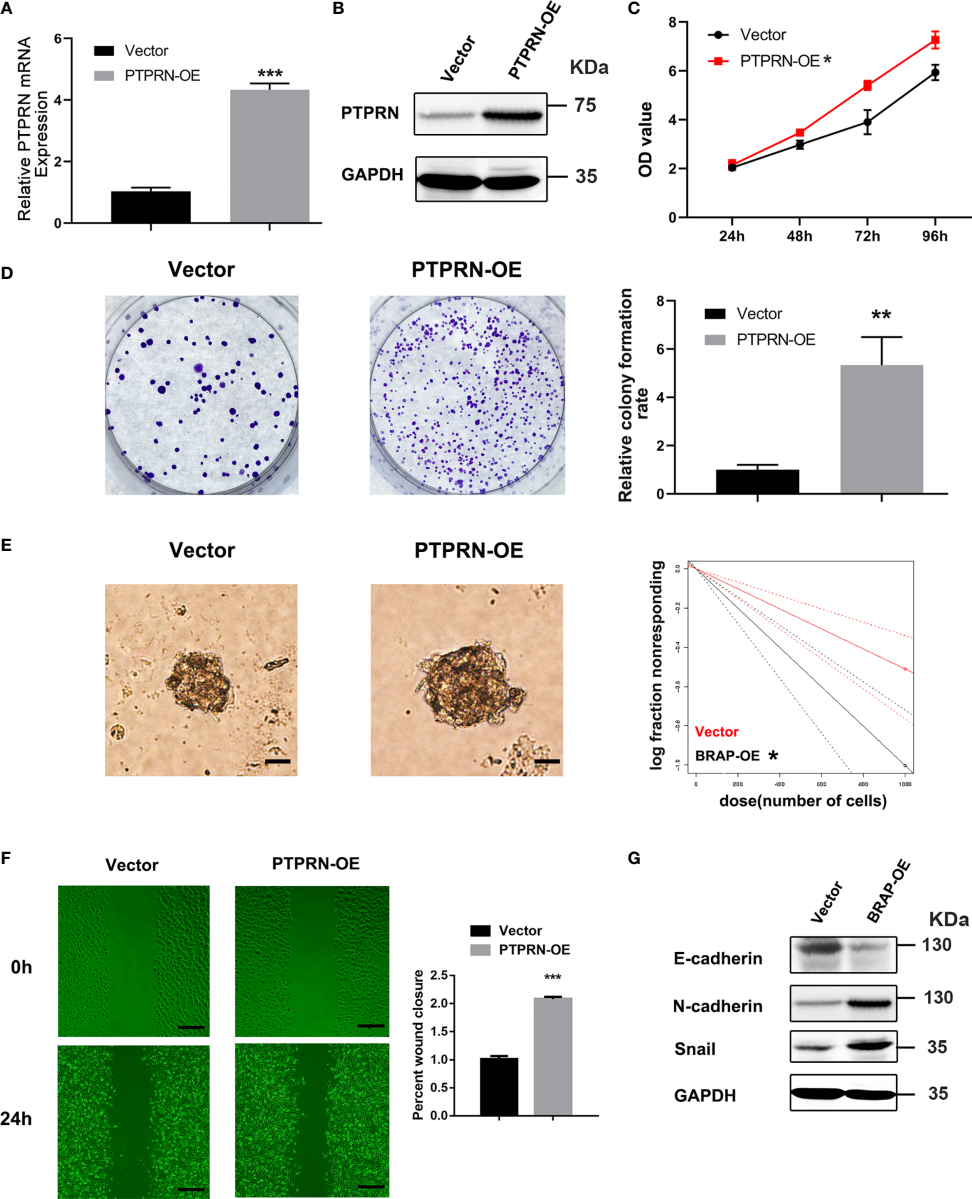
PTPRN overexpression induced the proliferation and migration of U251 cells. **(A, B)** The level of PTPRN was assayed by RT-qPCR and western blot analysis after the transfection with PTPRN overexpression plasmid; **(C)** The proliferation of the cells was detected by CCK-8; the corresponding OD values were detected at 24 h, 48 h, 72 h, and 96 h; **(D)** The proliferation of the cells was detected by colony-formation assays; the results were recorded using a camera at 14 days; **(E)** Sphere formation detected by microscope and calculated by ELDA after 14 days; **(F)** Cell migration detected by wound healing assay; the scratch was imaged at 0 and 24 h; The area of scratch reduction was calculated; **(G)** EMT-related protein (E-cadherin, N-cadherin, and Snail) expression in PTPRN-overexpression U251cells; scale bars, 100 μm; *p < 0.05, **p < 0.01, ***p < 0.001.

The results of MTS and colony formation assays demonstrated that upregulation of PTPRN induced the proliferation of U251 cells (**Figures 3A–D**). The migration was induced in U251 cells with high expression of PTPRN (**Figure 3E**). The glioma stem cell frequency was decreased in the PTPRN overexpression group (**Figure 3F**). The detection of epithelial mesenchymal transition (EMT)-related markers by western blot analysis showed that N-cadherin and Snail expression followed the trend of PTPRN. However, the level of E-cadherin was decreased after overexpression of PTPRN (**Figure 3G**).

### Effect of PTPRN Knockdown on the Transcriptome of U87 Glioma Cells

**Figure 4:**
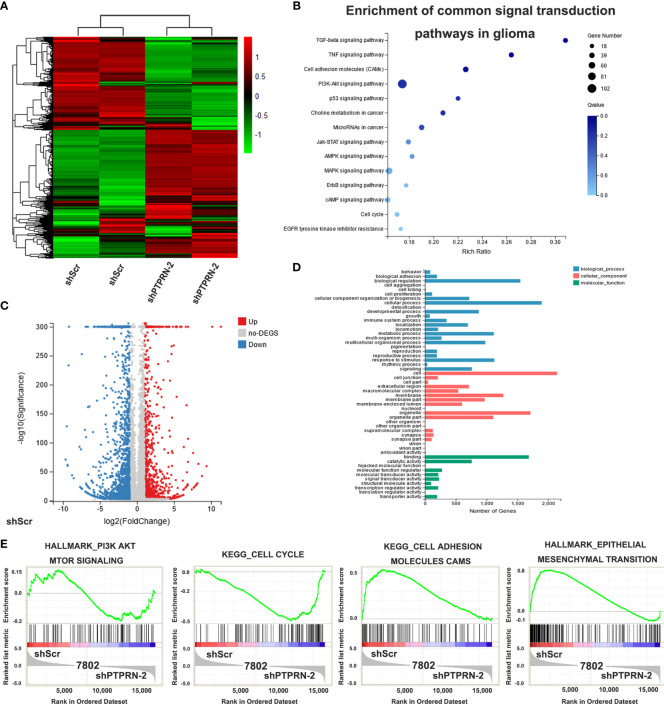
Effect of PTPRN knockdown on the transcriptome of U87 glioma cells. **(A)** Heat map of differentially expressed genes in shPTPRN U87 glioma cells; red represents highly expressed genes, and the green represents genes with low expression; the expression level is directly proportional to the color depth; **(B)** the KEGG method was used to analyze the differential genes and to select the enrichment pathways; **(C)** Volcano plot analysis; red represents highly expressed genes, and blue represents genes with low expression; the expression level is directly proportional to the color depth; **(D)** Gene Ontology analysis showed differential genes were categorized into three groups, including biological process, cellular component and molecular function; **(E)** GSEA was used to select the enrichment pathways.

RNA-seq was conducted to confirm the pathways that played a role in PTPRN functions in glioma. Fold change ≥2.0 and FDR ≤0.001 were used to identify the differential genes (**Figure 4A**). Then, the KEGG method was used to analyze the differential genes. PI3K/AKT pathway was also associated with the changes in PTPRN (**Figure 4B**). The number of genes with altered expression was shown by Volcano plot analysis (**Figure 4C**). We conducted Gene Ontology analysis, and differential genes were categorized into three groups, including biological process, cellular component and molecular function (**Figure 4D**). GSEA showed that the PIK3/AKT pathways were related to the changes in PTPRN (**Figure 4E**).

### PTPRN Knockdown Decreased Tumor Growth in a Mouse Xenograft Model

**Figure 5:**
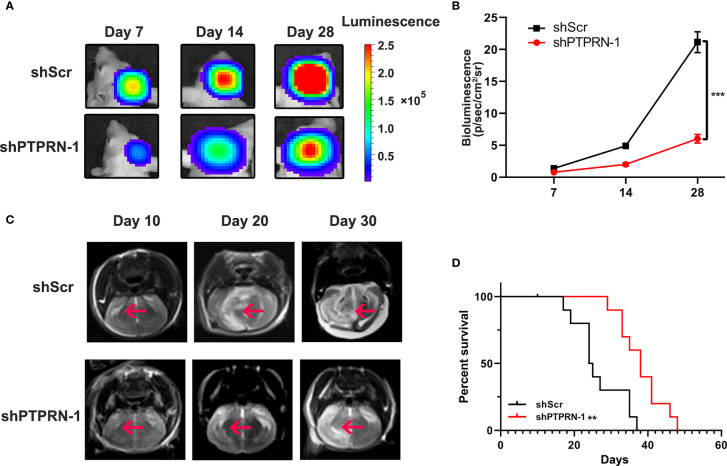
PTPRN knockdown decreased tumor growth in a mouse xenograft model. **(A)** Representative image of the tumor volume measured by bioluminescence imaging; **(B)** The intensity of fluorescence penetration into the brain tumor; **(C)** Representative image of the tumor volume measured by MRI; **(D)** Survival analysis of PTPRN expression in 12 intracranially implanted nude mice; Kaplan-Meier survival curves were used to analysis the difference of prognosis between the control group and PTPRN knockdown group; **p < 0.01, ***p < 0.001.

The volume of the tumor was measured by bioluminescence imaging (**Figure 5A**). The tumor volume of PTPRN knockdown mice was significantly decreased (**Figure 5B**). MRI also showed knockdown of PTPRN repressed the growth of the tumors (**Figure 5C**). Kaplan-Meier survival curves was used to analysis the difference of prognosis between control group and PTPRN knockdown group. The survival in the mouse xenograft model was improved by downregulation of PTPRN (**Figure 5D**).

### PTPRN Activated the PI3K/AKT Pathway by Interacting With Heat Shock Protein 90 Alpha Family Class A Member 1 (HSP90AA1)

**Figure 6:**
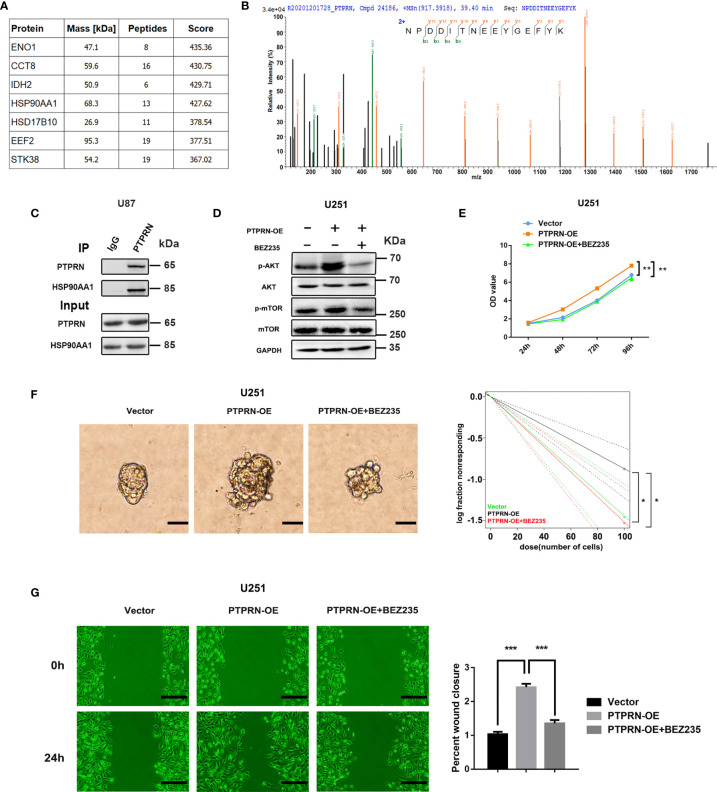
PTPRN activated the PI3K/AKT pathway by interacting with heat shock protein 90 alpha family class A member 1 (HSP90AA1). **(A)** Differentially expressed proteins interacting with PTPRN were identified by Co-IP and LC-MS/MS from FLAG-HA-PTPRN-expressing 293T cells, including an interaction between PTPRN and HSP90AA1. **(B)** Secondary spectrogram of target peptide showed PTPRN bind with HSP90AA1 directly. **(C)** Immunoprecipitation and western blotting demonstrated that PTPRN interacted with HSP90AA1 in U87 cells; **(D)** BEZ235 was used to reverse PI3K/AKT pathway activity in U251 cells with overexpression of PTPRN. AKT, p-AKT, mTOR, and p-mTOR expression were shown by western blot; **(E)** Cell proliferation detected by CCK-8; the corresponding OD values were detected at 24 h, 48 h, 72 h and 96 h; **(F)** Sphere formation detected by microscope and calculated by ELDA after 14 days; **(G)** The migration of the cells detected by wound healing assay; the scratch was imaged at 0 and 24 h; The area of scratch reduction was calculated; scale bars, 100 μm; *p < 0.05, **p < 0.01, ***p < 0.001.

An interaction between PTPRN and HSP90AA1 was identified by Co-IP and LC-MS/MS in FLAG-HA-PTPRN-expressing 293T cells (**Figure 6A**). Immunoprecipitation and western blot analysis demonstrated that PTPRN interacted with HSP90AA1 in U87 cells (**Figure 6B**). PTPRN regulated the PI3K/AKT pathway activity. BEZ235 was used to reverse the changes in the activity of the PI3K/AKT pathway in U251 cells induced by overexpression of PTPRN (**Figure 6C**). Treatment with BEZ235 reversed the growth and migration of PTPRN-overexpressing U251 glioma cells (**Figures 6D–G**).

## Discussion

Increasing evidence has shown that the PTP family does not always antagonize the activity of PTKs in regulating tyrosine phosphorylation and plays a crucial role in the initiation and progression of signalling cascades regulating cell functions, which cause many human diseases, such as cancer, metabolic syndrome and autoimmune diseases (12–14). The function and mechanism of action of PTPRN were investigated in certain tumors. These studies focused on the relationship between protein or mRNA expression and clinical prognosis, including hepatocellular carcinoma (15), lung cancer and other neuroendocrine tumors (16), ovarian cancer (17). In glioma research, Shergalis A et al. showed that PTPRN overexpression was strongly associated with poor overall survival in glioblastoma patients (18). Xu P et al. identified high PTPRN level as a crucial prognostic factor in glioma by weighted gene coexpression network analysis (WGCNA) (19). These study showed that PTPRN may play an important role in the occurrence and development of glioma; however, at present, there are no relevant experimental studies of PTPRN *in vivo* or *in vitro* in glioma.

The results of the present study indicated that a low level of PTPRN was associated with an improved prognosis in glioma patients. The results of clinical experiments were further verified by cell phenotype experiment. PTPRN expression was closely related to MGMT. MGMT is a unique DNA repair enzyme. MGMT removed O^6^-MG from DNA, protect tumor cells from the damage of alkylating agents, and then lead to temozolomide resistance of tumor cells, which has become an important factor restricting the effect of glioma chemotherapy. PI3K/AKT pathway is involved in the regulation of MGMT (20, 21). At present, the US Food and Drug Administration has granted a fast track for paxalisib, a PI3K inhibitor, to treat newly diagnosed glioblastoma patients with unmethylated MGMT gene promoter, who have completed preliminary radiotherapy and temozolomide treatment. Interestingly, we explored the mechanism by which PTPRN functions in glioma cells. GSEA showed that the PI3K/AKT pathway was also associated with the changes in PTPRN. The PI3K-Akt pathway is involved in the growth and apoptosis of cells. The activation of a variety of growth factors, hormones, and cytokines will lead to the further activation of this pathway, leading to the loss of normal differentiation and apoptosis of cells, leading to the occurrence of tumors (22). Notably, PI3K/AKT induced the accumulation of β-catenin in the nucleus, upregulated the expression of the downstream genes, and promoted the progression of epithelial-mesenchymal transition (EMT) (23). Epithelial-mesenchymal transition (EMT) is a biological process in which epithelial cells transform into cells with mesenchymal phenotype through specific procedures. E-cadherin can maintain tight junctions between cells and prevent cell invasion, metastasis and diffusion. N-cadherin is the main structural component of intercellular adhesion, and its main function is to mediate cell adhesion and migration. Snail, a transcription factor, binds to promoter regions such as E-cadherin to inhibit its transcription and promote epithelial-mesenchymal transition (24). In our experiment, PTPRN facilitated N-cadherin and Snail expression and inhibited E-cadherin expression, leading to tumor invasion and metastasis.

The results of a Co-IP experiment confirmed by mass spectrometry and Western blotting indicated that PTPRN regulated the activity of the PI3K/AKT pathway by interacting with HSP90AA1. HSP90AA1 is a member of the HSP90 family and one of the most important heat shock proteins. HSP90AA1 promotes the maturation, structural maintenance and proper regulation of specific target proteins and regulates cell cycle control and signal transduction. Some studies demonstrated that HSP90AA1 is closely related to a variety of malignant tumors (25, 26). A large-scale, multicentre, cross-validation clinical study demonstrated that plasma HSP90α can be used as a pan-cancer biomarker (27). HSP90AA1 stabilizes and activates c-Myc and participates in the occurrence of colorectal carcinoma and hepatocellular carcinoma (28, 29). Xiao X et al. demonstrated that HSP90AA1 promotes autophagy through the PI3K/Akt/mTOR pathway in osteosarcoma (30). Song KH et al. demonstrated that Nanog-activated HSP90A/AKT pathway is a potential target for immune refractory tumors (31). Therefore, we hypothesized that PTPRN interacts with HSP90AA1 to partially activate the PI3K/AKT pathway. The PI3K inhibitor treatment recovered PI3K/AKT pathway activity and phenotype of cells with PTPRN knockdown. These findings confirmed that the HSP90AA1/Akt signalling pathway may be involved in the proliferation and metastasis of glioma induced by PTPRN.

Thus, we suggest that glioma patients with high expression of PTPRN have a poor prognosis. PTPRN is an important proliferation- and metastasis-promoting factor. Furthermore, PTPRN may partially activate the PI3K/Akt pathway to influence the cell phenotype through HSP90AA1. These results indicate that reducing the expression of PTPRN in glioma cells can be used as a potential therapeutic strategy.

## Data Availability Statement

The datasets presented in this study can be found in online repositories. The names of the repository/repositories and accession number(s) can be found below: GEO Submission (GSE175831), https://www.ncbi.nlm.nih.gov/geo/query/acc.cgi?acc=GSE175831.

## Ethics Statement

The studies involving human participants were reviewed and approved by Ethics Committee of Tianjin Huanhu Hospital. The patients/participants provided their written informed consent to participate in this study. The animal study was reviewed and approved by Tianjin Huanhu hospital Animal Use and Care Committee.

## Author Contributions

DW, FT, and XL carried out most of the experimental work and the analysis of data. YF and YZ completed parts of the pathological experiment and uploaded the GEO data. HZ, JZ, BC, and BW provided scientific and administrative oversight for the conduct of the research and revised the manuscript. All authors contributed to the article and approved the submitted version.

## Funding

This work was supported by Chinese National Natural Science Foundation [grant number 82103247, 82171359, 81902477]; Tianjin Jinnan District Science and technology plan project [grant number 20200110]; Beijing Tianjin Hebei basic research cooperation project [grant number 19JCZDJC64600(Z)]; Science and Technology Development Foundation of Henan Province [212102310115, 212102310135]; Henan Provincial Medical Science and Technology Project [SBGJ202002025, SBGJ202003009]; Henan Medical Science and Technology Innovation Talent Project [YXKC2020045].

## Conflict of Interest

The authors declare that the research was conducted in the absence of any commercial or financial relationships that could be construed as a potential conflict of interest.

## Publisher’s Note

All claims expressed in this article are solely those of the authors and do not necessarily represent those of their affiliated organizations, or those of the publisher, the editors and the reviewers. Any product that may be evaluated in this article, or claim that may be made by its manufacturer, is not guaranteed or endorsed by the publisher.

## Supplementary Material

Supplementary Figure 1 The expression of VEGF and MGMT by immunohistochemistry staining in high-grade glioma. The expression of VEGF and MGMT detected by immunohistochemistry staining in high-grade glioma (negative and positive).
