# Supplementary figures and images for: Expression and Tumor-Promoting Effect of Tyrosine Phosphatase Receptor Type N (PTPRN) in Human Glioma
Source: Front Oncol. 2021 Sep 7;11:676287. doi: 10.3389/fonc.2021.676287 (PMC8453168; doi:10.3389/fonc.2021.676287)

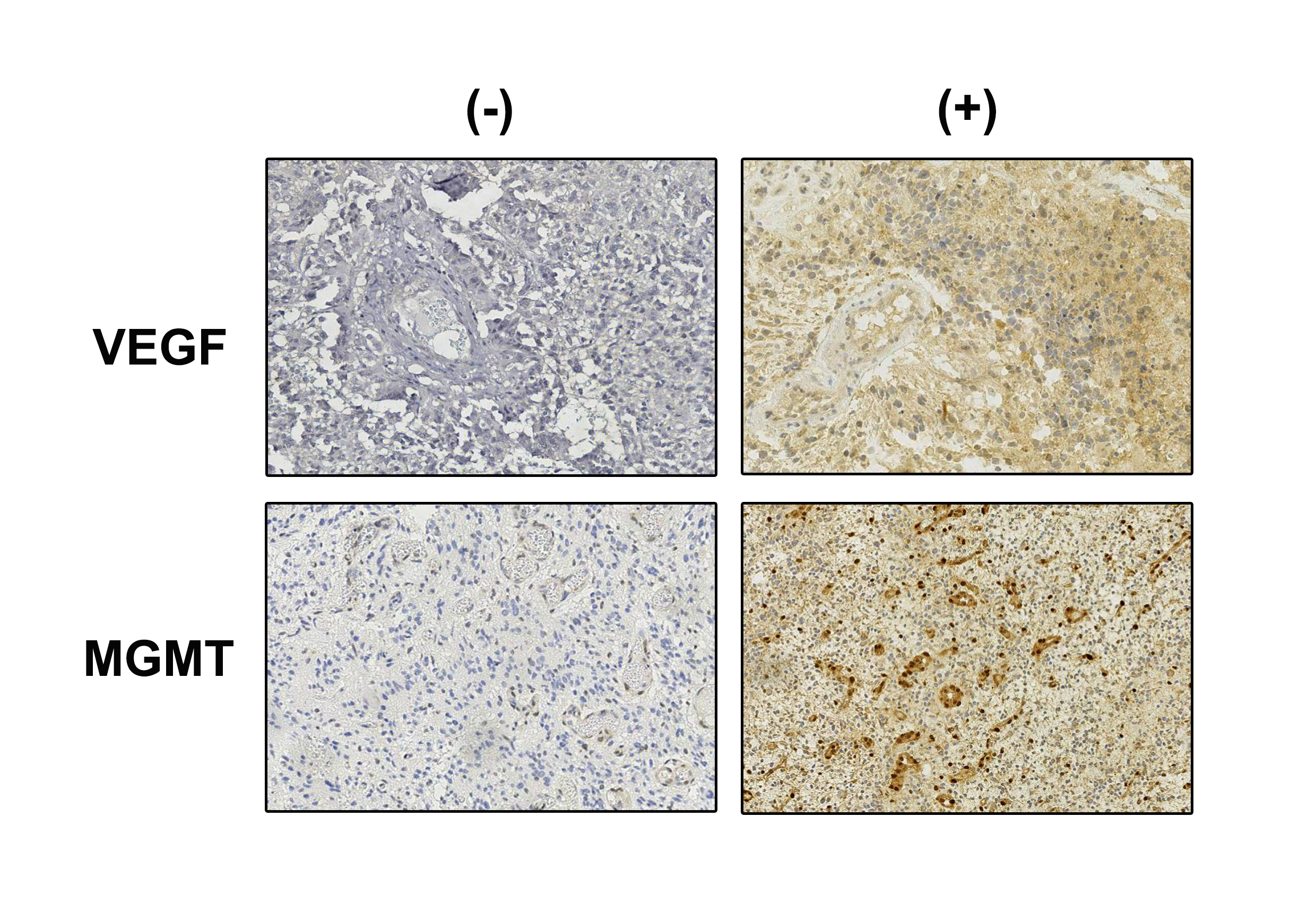

Supplement: Supplementary Figure 1 — The expression of VEGF and MGMT by immunohistochemistry staining in high-grade glioma. The expression of VEGF and MGMT detected by immunohistochemistry staining in high-grade glioma (negative and positive). [file Image_1.tif]
