# Supplementary material for: Expression and Tumor-Promoting Effect of Tyrosine Phosphatase Receptor Type N (PTPRN) in Human Glioma
Source: Front Oncol. 2021 Sep 7;11:676287. doi: 10.3389/fonc.2021.676287 (PMC8453168; doi:10.3389/fonc.2021.676287)
Supplement: Supplementary file 2 [file Table_1.docx]

Supplementary Material

# Supplementary Table 1 Primer sequences

|  | **shRNA Oligonucleotides** |
| --- | --- |
| **shPTPRN-1** | 5'-AAGGTGTGCTCCGACAACTCA-3' |
| **shPTPRN-2** | 5'-AACTGCCTCCAAGGGCATATT-3' |
| **shScramble** | 5'-CCTAAGGTTAAGTCGCCCTCG-3' |
|  | **Primers used for cloning** |
| **PTPRN** overexpression | forward: ATGCGGCGCCCGCGGCGGCCT |
|  | reverse: TCACTGGGGCAGGGCCTTGAGG |
|  | Primer used for qPCR |
| **PTPRN** | forward:5'-CCACCCTTCACTGAGTTACGA-3' |
|  | reverse:5'-AAATATGCCCTTGGAGGCAGT-3' |
| **GAPDH** | forward:5'-GCACCGTCAAGGCTGAGAAC-3' |
|  | reverse:5'-TGGTGAAGACGCCAGTGGA-3' |
